# Essential Guide to Manuscript Writing for Academic Dummies: An Editor's Perspective

**DOI:** 10.1155/2022/1492058

**Published:** 2022-09-01

**Authors:** Syed Sameer Aga, Saniya Nissar

**Affiliations:** ^**1**^ Department of Basic Medical Sciences, Quality Assurance Unit, College of Medicine, King Saud bin Abdulaziz University for Health Sciences (KSAU-HS), King Abdullah International Medical Research Center (KAIMRC), Ministry of National Guard Health Affairs (MNGHA), King Abdulaziz Medical City, Jeddah 21423, Saudi Arabia; ^2^Molecular Diseases & Diagnostics Division, Infinity Biochemistry Pvt. Ltd, Sajad Abad, Chattabal, Srinagar, Kashmir 190010, India

## Abstract

Writing an effective manuscript is one of the pivotal steps in the successful closure of the research project, and getting it published in a peer-reviewed and indexed journal adds to the academic profile of a researcher. Writing and publishing a scientific paper is a tough task that researchers and academicians must endure in staying relevant in the field. Success in translating the benchworks into the scientific content, which is effectively communicated within the scientific field, is used in evaluating the researcher in the current academic world. Writing is a highly time-consuming and skill-oriented process that requires familiarity with the numerous publishing steps, formatting rules, and ethical guidelines currently in vogue in the publishing industry. In this review, we have attempted to include the essential information that novice authors in their early careers need to possess, to be able to write a decent first scientific manuscript ready for submission in the journal of choice. This review is unique in providing essential guidance in a simple point-wise manner in conjunction with easy-to-understand illustrations to familiarize novice researchers with the anatomy of a basic scientific manuscript.

## 1. Background

Communication is the pivotal key to the growth of scientific literature. Successfully written scientific communication in the form of any type of paper is needed by researchers and academicians alike for various reasons such as receiving degrees, getting a promotion, becoming experts in the field, and having editorships [1, 2].

Here, in this review, we present the organization and anatomy of a scientific manuscript enlisting the essential features that authors should keep in their mind while writing a manuscript.

## 2. Types of Manuscripts

**Figure 1:**
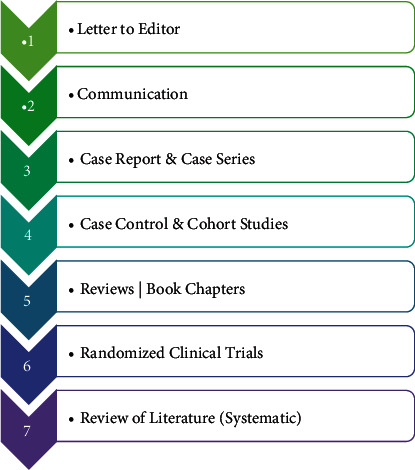
Types of manuscripts based on complexity of content and context.

Numerous types of manuscripts do exist, which can be written by the authors for a possible publication (Figure 1). Primarily, the choice is dependent upon the sort of communication authors want to make. The simplest among the scientific manuscripts is the “Letter to an Editor,” while “Systematic Review” is complex in its content and context [3].

## 3. Anatomy of the Manuscript

Writing and publishing an effective and well-communicative scientific manuscript is arguably one of the most daunting yet important tasks of any successful research project. It is only through publishing the data that an author gets the recognition of the work, gets established as an expert, and becomes citable in the scientific field [4]. Among the numerous types of scientific manuscripts which an author can write (Figure 1), original research remains central to most publications [4–10].

**Figure 2:**
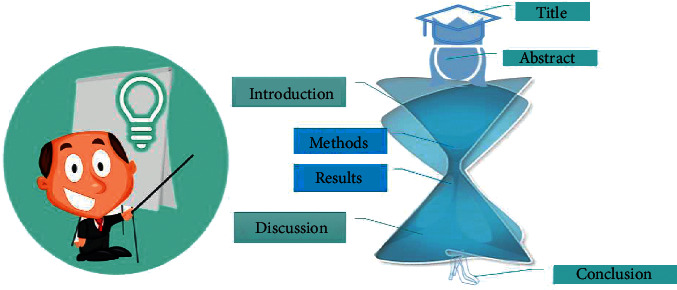
Generalized anatomy of manuscript based on IMRAD format.

**Figure 3:**
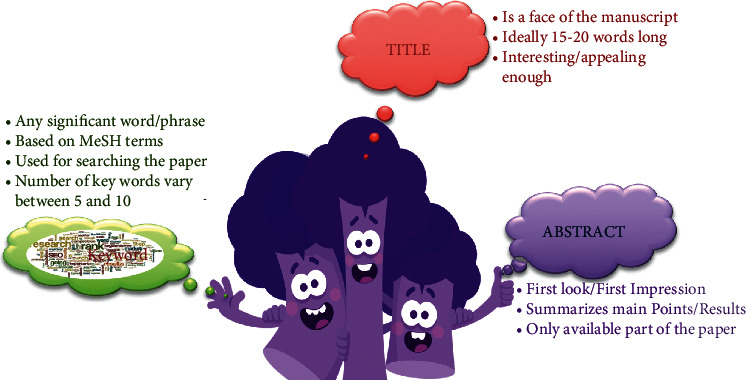
Three important contents of the title page—title, abstract, and keywords.

A good scientific paper essentially covers the important criteria, which define its worth such as structure, logical flow of information, content, context, and conclusion [5]. Among various guidelines that are available for the authors to follow, IMRAD scheme is the most important in determining the correct flow of content and structure of an original research paper [4, 11–13]. IMRAD stands for introduction, methods, results, and discussion (Figure 2). Besides these, other parts of the manuscript are equally essential such as title, abstract, keywords, and conclusion (Figure 3).

IMRAD scheme was introduced in the early 1900 by publishers to standardize the single format of the scientific manuscript and since then is the universal format used by most the publishing houses [6, 14–17]. In the next sections, the contents and criteria of each of them are explained in detail. A list of the most common mistakes, which the author makes in these sections, is also provided in the tabulated form [18] (Table 1).

## 4. Title

- The title is the most important element of the paper, the first thing readers encounter while searching for a suitable paper [1]. It reflects the manuscript's main contribution and hence should be simple, appealing, and easy to remember [7].
- A good title should not be more than 15 words or 100 characters. Sometimes journals ask for a short running title, which should essentially be no more than 50% of the full title. Running titles need to be simple, catchy, and easy to remember [19, 20].
- Keeping the titles extremely long can be cumbersome and is suggestive of the authors' lack of grasp of the true nature of the research done.
- It usually should be based on the keywords, which feature within the main rationale and/or objectives of the paper. The authors should construct an effective title from keywords existing in all sections of the main text of the manuscript [19].
- Having effective keywords within the title helps in the easy discovery of the paper in the search engines, databases, and indexing services, which ultimately is also reflected by the higher citations they attract [1].
- It is always better for the title to reflect the study's design or outcome [21]; thus, it is better for the authors to think of a number of different titles proactively and to choose the one, which reflects the manuscript in all domains, after careful deliberation. The paper's title should be among the last things to be decided before the submission of the paper for publication [20].
- Use of abbreviations, jargons, and redundancies such as “a study in,” “case report of,” “Investigations of,” and passive voice should be avoided in the title.

## 5. Abstract

- The abstract should essentially be written to answer the three main questions—“What is new in this study?” “What does it add to the current literature?” and “What are the future perspectives?”
- A well-written abstract is a pivotal part of every manuscript. For most readers, an abstract is the only part of the paper that is widely read, so it should be aimed to convey the entire message of the paper effectively [1].
- Depending upon the journal, an abstract's word count can range from 70 to 300 words [7] and can be either structured or nonstructured in its presentation (Figure 4).
- An effective abstract is a rationalized summary of the whole study and essentially should contain well-balanced information about six things: background, aim, methods, results, discussion, and conclusion [6, 19].
- A good abstract is succinct, easy to read, and independent in carrying the information. Care should be taken in providing the three Cs—context, content, and conclusion (C-C-C) (Figure 5) while writing it. Context provides the “gap in the literature,” content covers the “what has been done in this study,” and the conclusion provides the “single take-home message of the study” [1]. An effective abstract should be able to tell a complete story to the readers.
- An abstract should be written at the end, after finishing the writing of an entire manuscript to be able to stand-alone from the main text. It should reflect your study completely without any reference to the main paper [19].
- The authors need to limit/write their statements in each section to two or three sentences. However, it is better to focus on results and conclusions, as they are the main parts that interest the readers and should include key results and conclusions made thereof.
- Inclusion of excessive background information, citations, abbreviations, use of acronyms, lack of rationale/aim of the study, lack of meaningful data, and overstated conclusions make an abstract ineffective.

## 6. Keywords

- Keywords are the important words, which feature repeatedly in the study or else cover the main theme/idea/subject of the manuscript. They are used by indexing databases such as PubMed, Scopus, and Embase in categorizing and cross-indexing the published article.
- It is always wise to enlist those words which help the paper to be easily searchable in the databases.
- Keywords can be of two types: (a) general ones that are provided by the journal or indexing services called as medical subject headings (MeSH) as available in NCBI (https://www.ncbi.nlm.gov/mesh/) and (b) custom ones made by authors themselves based on the subject matter of the study [6, 20].
- Upon submission, journals do usually ask for the provision of five to ten keywords either to categorize the paper into the subject areas or to assign it to the subspecialty for its quick processing.

## 7. Introduction

(i) The whole idea of writing this section is to cover two important questions—“What are the gaps present in the current literature?” and “Why is the current study important?”
(ii) Introduction provides an opportunity for the authors to highlight their area of study and provide rationale and justification as to why they are doing it [20, 22, 23].
(iii) An effective introduction usually constitutes about 10–15% of the paper's word count [22].
(iv) It should generally be made up of four paragraphs, each dedicated to cover four specific questions in a funnel-shaped manner, traversing from a more generalized view to the specific details (Figure 6) [1, 6, 13, 20, 24].
  1. The first paragraph of the introduction should always cover “What is known about the area of study?” or “What present/current literature is telling about the problem?” All relevant and current literature/studies, i.e., original studies, meta-analyses, and systematic reviews, should be covered in this paragraph.
  2. The second paragraph should cover “What is unknown or not done about this issue/study area?” The authors need to indicate the aspects of what has not been answered about the broader area of the study until now.
  3. The third paragraph should identify the gaps in the current literature and answer “What gaps in the literature would be filled by their current study?” This part essentially identifies the shortcoming of the existing studies.
  4. The fourth paragraph should be dedicated to effectively writing “What authors are going to do to fill the gaps?” and “Why do they want to do it?” This paragraph contains two sections—one explains the rationale of the study and introduces the hypothesis of the study in form of questions “What did authors do? and Why they did do so?” and the second enlists specific objectives that the authors are going to explore in this study to answer “Why this study is going to be important?” or “What is the purpose of this study?”.
(v) Introduction is regarded as the start of the storyline of manuscript, and hence, the three Cs' scheme (Figure 5) becomes more relevant while writing it: the context in terms of what has been published on the current idea/problem around the world, content as to what you are going to do about the problem in hand (rationale), and conclusion as to how it is going to be done (specific objective of the study) [1, 23].
(vi) Introduction is the first section of the main manuscript, which talks about the story; therefore, while writing it authors should always try to think that “would this introduction be able to convince my readers?” [25]. To emphasize on the importance of the study in filling the knowledge gap is pivotal in driving the message through [23].
(vii) Introduction should never be written like a review, any details, contexts, and comparisons should be dealt within the discussion part [16].
(viii) While choosing the papers, it is wise to include the essential and recent studies only. Studies more than 10 years old should be avoided, as editors are inclined towards the recent and relevant ones only [20, 22].
(ix) In the last paragraph, enlisting the objectives has a good impact on readers. A clear distinction between the primary and secondary objectives of the study should be made while closing the introduction [22].

## 8. Methods

(i) It is regarded as the skeleton of the manuscript as it contains information about the research done. An effective methods section should provide information about two essential aspects of the research—(a) precise description of how experiments were done and (b) rationale for choosing the specific experiments.
(ii) It essentially constitutes 30–40% of the manuscript and should carry exhaustive description of seven areas of the study (Figure 7).
  1. Study Settings: describing the area or setting where the study was conducted. This description should cover the details relevant to the study topic.
  2. Study Design: identifying the study as cross-sectional, case-control, intervention study, etc. Specific guidelines to write manuscripts according to universal guideline are also needed to be followed (Table 2).
  3. Sample Size and Sampling Technique: mentioning what number of samples is needed and how they would be collected.
  4. Ethical Approvals: clearly identifying the study approval body or board and proper collection of informed consent from participants.
  5. Recruitment Methods: using at least three criteria for the inclusion or exclusion of the study subjects to reach an agreed sample size.
  6. Experimental and Intervention Details: exhaustively describing each and every detail of all the experiments and intervention carried out in the study for the readers to reproduce independently.
  7. Statistical Analysis: mentioning all statistical analysis carried out with the data which include all descriptive and inferential statistics and providing the analysis in meaningful statistical values such as mean, median, percent, standard deviation (SD), probability value (p), odds ratio (OR), and confidence interval (CI).
(iii) Methods should be elaborative enough that the readers are able to replicate the study on their own. If, however, the protocols are frequently used ones and are already available in the literature, the authors can cite them without providing any exhaustive details [26].
(iv) Methods should be able to answer the three questions for which audience reads the paper—(1) What was done? (2) Where it was done? and (3) How it was done? [11].
(v) Remember, methods section is all about “HOW” the data were collected contrary to “WHAT” data were collected, which should be written in the results section. Therefore, care should be taken in providing the description of the tools and techniques used for this purpose.
(vi) Writing of the methods section should essentially follow the guidelines as per the study design right from the ideation of the project. There are numerous guidelines available, which author's must make use of, to streamline the writing of the methods section in particular (see Table xx for details).
(vii) Provision of the information of the equipment, chemicals, reagents, and physical conditions is also vital for the readers for replication of the study. If any software is used for data analysis, it is imperative to mention it. All manufacturer's names, their city, and country should also be provided [6, 11].

## 9. Results

- The purpose of the results section of the manuscript is to present the finding of the study in clear, concise, and objective manner to the readers [7, 27, 28].
- Results section makes the heart of the manuscript, as all sections revolve around it. The reported findings should be in concordance with the objectives of the study and be able to answer the questions raised in the introduction [6, 20, 27].
- Results should be written in past tense without any interpretation [6, 27].
- Results section mimics the methods section in chronological order of its contents and structure in a “Ying-Yang” scheme [27] (Figure 8). Breaking the contents into subheadings facilitates the readers to understand the data in an effective manner.
- It is always better to take refuge in tables and figures to drive the exhaustive data through. Repetition of the data already carried in tables, figures, etc., should be avoided [4, 6, 20].
- Proper positioning and citations of the tables and figures within the main text are also critical for the flow of information and quality of the manuscript [6, 11].
- Results section should carry clear descriptive and inferential statistics in tables and/or figures, for ease of reference to readers.
- Provision of the demographic data of the study participants takes priority in the results section; therefore, it should be made as its first paragraph. The subsequent paragraphs should introduce the inferential analysis of the data based on the rationale and objectives of the study. The last paragraphs mention what new results the study is going to offer [6, 11, 20].
- authors should not attempt to report all analysis of the data. Discussing, interpreting, or contextualizing the results should be avoided [20].

## 10. Discussion

(i) The main purpose of writing a discussion is to fill the gap that was identified in the introduction of the manuscript and provide true interpretations of the results [6, 11, 20].
(ii) The general structure of discussion follows the same pattern as in introduction, but in pyramid (reverse funnel-up) scheme covering the three things of content-context-conclusion (C-C-C) (Figure 9). The focus should be on the storyline at all times and to identify the pearls and pitfalls of the study.
(iii) Discussion section toggles between two things—content and context. The authors need to exhaustively describe their interpretation of the analyzed data (content) and then compare it with the available relevant literature (context) [1, 29]. Finally, it should justify everything in conclusion as to what all this means for the field of study.
(iv) The comparison can either be concordant or discordant, but it needs to highlight the uniqueness and importance of the study in the field. Care should be taken not to cover up any deviant results, which do not gel with the current literature [30].
(v) In discussion it is safe to use words such as “may,” “might,” “show,” “demonstrate,” “suggest,” and “report” while impressing upon your study's data and analyzed results.
(vi) Putting results in context helps in identifying the strengths and weakness of the study and enables readers to get answers to two important questions—one “what are the implications of the study?” Second “how the study advance the field further?” [1, 30].
(vii) Essentially, it should contain 20% of the word count of the manuscript and consist of five to eight paragraphs [4, 20, 29].
  1. The first paragraph of the discussion is reserved for highlighting the key results of the study as briefly as possible [4, 6]. However, care should be taken not to have any redundancy with the results section. The authors should utilize this part to emphasize the originality and significance of their results in the field [1, 4, 11, 20].
  2. The second paragraph should deal with the importance of your study in relationship with other studies available in the literature [4].
  3. Subsequent paragraphs should focus on the context, by describing the findings in comparison with other similar studies in the field and how the gap in the knowledge has been filled [1, 4].
  4. In the penultimate paragraph, authors need to highlight the strengths and limitations of the study [4, 6, 30].
  5. Final paragraph of the discussion is usually reserved for drawing the generalized conclusions for the readers to get a single take-home message.
(viii) A well-balanced discussion is the one that effectively addresses the contribution made by this study towards the advancement of knowledge in general and the field of research in particular [7]. It essentially should carry enough information that the audience knows how to apply the new interpretation presented within that field.

## 11. Conclusion

- It usually makes the last part of the manuscript, if not already covered within the discussion part [6, 20].
- Being the last part of the main text, it has a long-lasting impact on the reader and hence should be very clear in presenting the chief findings of the paper as per the rationale and objectives of the study [4, 20].
- A conclusion should be terse in communicating three things to the reader—what is the memorable take-home message, what new findings have been added to the literature, and what are the future perspectives in the field [4, 6, 11] (Figure 10).

## 12. References or Bibliography

- Every article needs a suitable and relevant citation of the available literature to carry the contextual message of their results to the readers [31].
- Inclusion of proper references in the required format, as asked by the target journal, is necessary.
- Numerous styling formats of citations are available in the scientific world such as APA, AMA, NLM, and MLA, and usually, each publishing house has its own preference (Figure 11).
- Depending upon the journal and publishing house, usually, 30–50 citations are allowed in an original study, and they need to be relevant and recent.

## 13. Organization of the Manuscript Package

**Figure 4:**
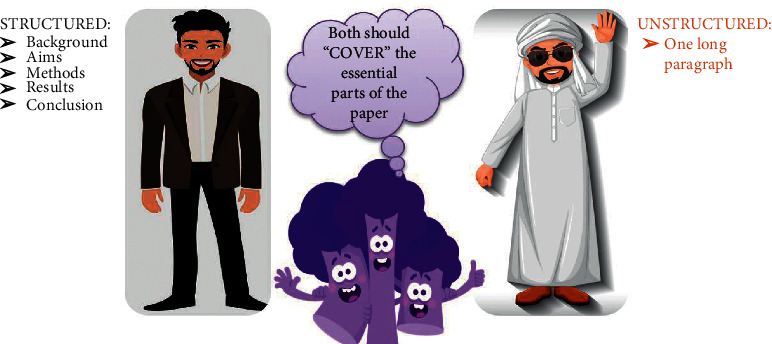
Two major types of abstract—structured and unstructured. Structured abstracts are piecemealed into five different things, each consisting of one or two sentences, while unstructured abstracts consist of single paragraph written about the same things.

**Figure 5:**
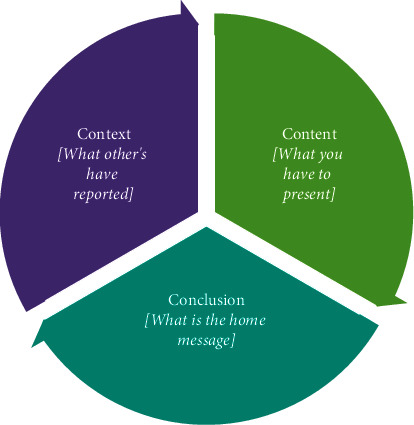
Three C concept followed while writing the manuscript.

**Figure 6:**
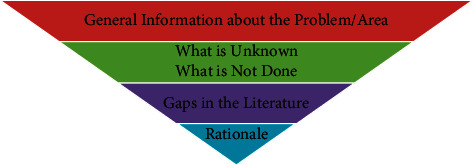
Funnel-down scheme followed while writing the introduction section of manuscript, moving from broader to specific information.

**Figure 7:**
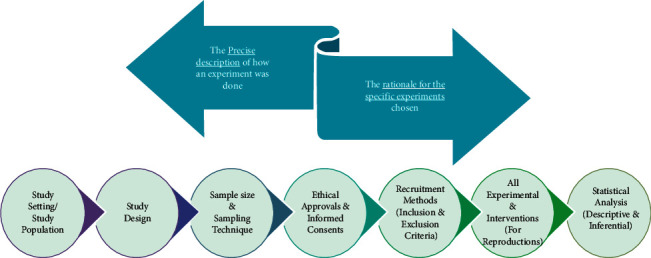
Methods and the seven areas which it should exhaustively describe.

**Figure 8:**
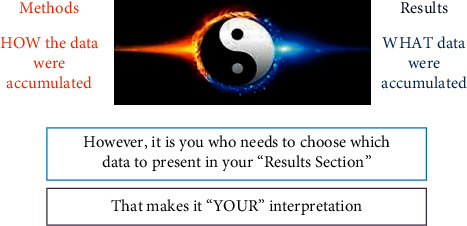
Interdependence between methods and results of the manuscript.

**Figure 9:**
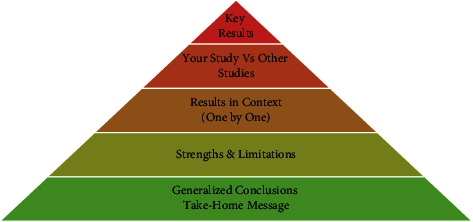
Pyramid scheme followed while writing the discussion section of manuscript, moving from the key results of the study to the specific conclusions.

**Figure 10:**
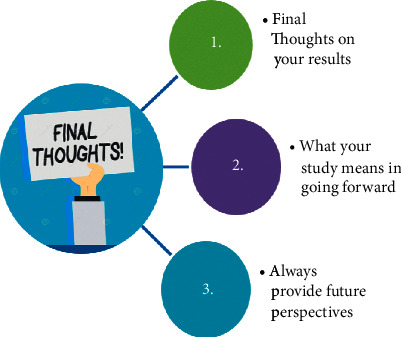
Crux of the conclusion section.

**Figure 11:**
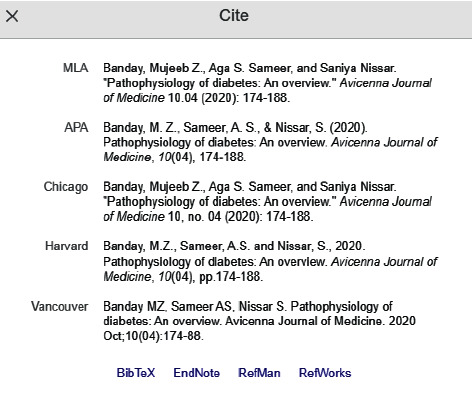
A Google Scholar screenshot of different styles of formatting of references.

Ideally, all manuscripts, no matter where they have to be submitted, should follow an approved organization, which is universally used by all publication houses. “Ready to submit” manuscript package should include the following elements:

(i) Cover letter, addressed to the chief editor of the target journal.
(ii) Authorship file, containing the list of authors, their affiliations, emails, and ORCIDs.
(iii) Title page, containing three things—title, abstract, and keywords.
(iv) Blinded main manuscript, constituting of:
  1. Main text structured upon IMRAD scheme.
  2. References as per required format.
  3. Legends to all tables and figures.
  4. Miscellaneous things such as author contributions, acknowledgments, conflicts of interest, funding body, and ethical approvals.
(v) Tables as a separate file in excel format.
(vi) Figures or illustrations, each as a separate file in JPEG or TIFF format [32].
(vii) Reviewers file, containing names of the suggested peer reviewers working or publishing in the same field.
(viii) Supplementary files, which can be raw data files, ethical clearance from Institutional Review Board (IRBs), appendixes, etc.

## 14. Overview of an Editorial Process

**Figure 12:**
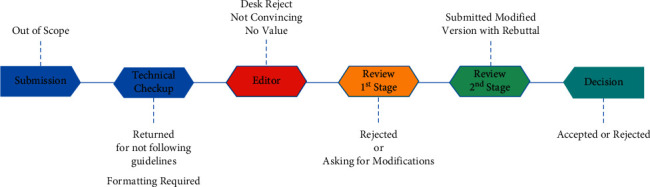
An overview of the journal's editorial process.

Each scientific journal has a specific publication policies and procedures, which govern the numerous steps of the publication process. In general, all publication houses process the submission of manuscripts via multiple steps tightly controlled by the editors and reviewers [33].  Figure 12 provides general overview of the six-step editorial process of the scientific journal.

## 15. Summary

The basic criteria for writing any scientific communication are to know how to communicate the information effectively. In this review, we have provided the critical information of do's and don'ts for the naive authors to follow in making their manuscript enough impeccable and error-free that on submission manuscript is not desk rejected at all. but this goes with mentioning that like any other skill, and the writing is also honed by practicing and is always reflective of the knowledge the writer possesses. Additionally, an effective manuscript is always based on the study design and the statistical analysis done. The authors should always bear in mind that editors apart from looking into the novelty of the study also look at how much pain authors have taken in writing, following guidelines, and formatting the manuscript. Therefore, the organization of the manuscript as per provided guidelines such as IMRAD, CONSORT, and PRISMA should be followed in letter and spirit. Care should be taken to avoid the mistakes, already enlisted, which can be the cause of desk rejection. As a general rule, before submission of the manuscript to the journal, sanitation check involving at least two reviews by colleagues should be carried out to ensure all general formatting guidelines are followed.

## Figures and Tables

**Table 1:** Common mistakes authors make in their manuscripts.

Section of manuscript	Common mistakes
Title	(i) Too long
	(ii) Not consistent with subject and rationale of study
	(iii) Title not smart enough
	(iv) Use of abbreviations, acronyms, and jargons

Abstract	(i) Longer than prescribed word count
	(ii) Not effectively stratified section wise
	(iii) Essentially copy-pasted from main text
	(iv) Contains information not present in main paper
	(v) Citations included
	(vi) No effective take-home message
	(vii) Written as introduction or conclusion of the paper

Keywords	(i) Missing essential keywords
	(ii) No MeSH terms used
	(iii) Insufficient numbers in manuscript
	(iv) Wrong keywords not related to subject used
	(v) Abbreviations used

Introduction	(i) Overshooting the prescribed word count in detail (>15%)
	(ii) No identification of context, content, and conclusions
	(iii) Not citing recent and relevant research
	(iv) Deliberate omission of contradictory studies
	(v) Rationale, aim, and objectives of research not indicated

Methods	(i) Type of the study not indicated
	(ii) Study settings—location, period, dates, etc., not revealed
	(iii) Inclusion and exclusion criteria for participants not provided
	(iv) Lack of sample size and sampling technique descriptions
	(v) Ethical clearance of the study not provided
	(vi) Absence of informed consent from participants
	(vii) Exhaustive replicative details of the experiments not provided
	(viii) No validated experiments, questionnaires, or instruments used
	(ix) No clear mention of statistical analysis used
	(x) Statistical significance not set

Results	(i) Results written in present tense
	(ii) Results not related to the objectives of the study mentioned
	(iii) Redundancy with methods section
	(iv) Incorrect statistical tests used
	(v) Overlapping the information present in figures and tables
	(vi) Unnecessary citations incorporated
	(vii) Stratified and biased use of data
	(viii) Wrong interpretation of statistical analysis
	(ix) Missing essential details of the analyzed data
	(x) Missing data and values in the tables
	(xi) Measurement units not provided properly
	(xii) Multiple formats of the statistical significance used (*p*=0.05, 0.0001, 0.00, etc.)

Discussion	(i) Not all data present are discussed effectively
	(ii) Exacerbation of the results
	(iii) Nonsignificant results exhaustively discussed
	(iv) Insertion of new data not carried previously in results
	(v) Biased interpretations of analyzed data
	(vi) No regard of the context, content, and conclusion
	(vii) Outdated citations used for context (>10 years old)
	(viii) Strengths or limitations of the study not clearly mentioned
	(ix) Future prospects of the study not mentioned

Conclusion	(i) Overstated what the data reveal
	(ii) Vague and not supported by the data
	(iii) Too brief without any take-home message
	(iv) No essential connection with the objectives
	(v) Essential results of the study underscored
	(vi) No future perspectives of the study area provided

References	(i) Too many or too few citations than prescribed
	(ii) Too old studies included (>10 years old)
	(iii) Proper formatting of the citations not carried out
	(iv) Studies not related to field cited
	(v) Studies contradictory to results deliberately left out
	(vi) Too many self-citations made
	(viii) Citations in tables and figures not included

Others	(i) Headings and subheadings missing in the main text
	(ii) Logical flow of ideas not followed in main text
	(iii) Poor quality/low-resolution figures/illustrations provided
	(iv) Figures not in proper format (JPEG, TIFF, PNG, etc.)
	(v) Figure and table legends not provided
	(vi) Illustrations included within the main manuscript
	(vii) Tables and figures not cited within the main text
	(viii) Too many tables or figures used (>8 in number)
	(ix) Use of patients' pictures without the consent
	(x) Too much of plagiarism (>15%)
	(xi) Lack of information about authors' affiliations, official emails, and ORCID
	(xii) No mention of each author's contribution to the study/paper
	(xiii) Corresponding/submitting author not identified
	(xiv) Lack of declaration of conflicts
	(xv) No disclosure of financial/grant support

**Table 2:** Different guidelines available for perusal of the authors for writing an effective manuscript.

Guideline	Full form	Used for	URL
IMRaD	Introduction, Methods, Results, and Discussion	For all papers being submitted	https://www.ncbi.nlm.nih.gov/pmc/articles/PMC442179/
CONSORT	Consolidated Standards of Reporting Trials	For randomized controlled trials	https://www.consort-statement.org/consort-2010
TREND	Transparent Reporting of Evaluations with Nonrandomized Designs	For non-randomized trials	https://www.cdc.gov/trendstatement/
PRISMA	Preferred Reporting Items for Systematic Reviews and Meta-Analyses	For systematic review and meta-analyses	https://www.prisma-statement.org/
CARE	CAse REports	For case reports	https://www.care-statement.org/resources/checklist
STROBE	Strengthening the Reporting of Observational Studies in Epidemiology	For observational studies	https://www.strobe-statement.org/index.php?id=available-checklists
STREGA	STrengthening the REporting of Genetic Association Studies	For genetic association studies	https://www.equator-network.org/reporting-guidelines/strobe-strega/
SRQR	Standards for Reporting Qualitative Research	For qualitative studies	https://www.equator-network.org/reporting-guidelines/srqr/
STARD	Standards for Reporting of Diagnostic Accuracy Studies	For diagnostic accuracy studies	https://www.stard-statement.org/
ARRIVE	Animal Research Reporting of In Vivo Experiments	For animal experiments	https://www.nc3rs.org.uk/arrive-guidelines

## Data Availability

No data were used in this review.
